# Role of childhood trauma in psychogenic non-epileptic seizures: a report from China

**DOI:** 10.1186/s42494-024-00180-5

**Published:** 2025-01-06

**Authors:** Xin Tong, Rong Luo, Xiao Hu, Dong Zhou, Dongmei An

**Affiliations:** 1 Department of Neurology, West China Hospital, Sichuan University, No. 37 Guoxue Road, Chengdu, 610041 Sichuan China; 2 Department of Pediatrics, West China Second University Hospital, Sichuan University, Chengdu, 610041 China; 3 Key Laboratory of Birth Defects and Related Diseases of Women and Children, Ministry of Education, Sichuan University, Sichuan University, Chengdu, 610041 China; 4 Department of Rehabilitation Medicine, West China Second University Hospital, Sichuan University, Chengdu, 610041 China

**Keywords:** Psychogenic non-epileptic seizures, Risk factor, Childhood trauma, Mental health, Parent-child separation, Dissociative symptoms

## Abstract

**Background:**

To investigate the prevalence, risk factors, and impacts of childhood trauma in Chinese patients with psychogenic non-epileptic seizures (PNES) compared to the healthy population.

**Methods:**

Patients with PNES and sex- and age-matched healthy controls were recruited. All the participants were interviewed to collect demographics, information of childhood environment, and clinical characteristics. Each participant completed the Childhood Trauma Questionnaire-Short Form (CTQ-SF), the Dissociative Experiences Scale (DES), and the Symptom Checklist-90 (SCL-90). Factors associated with childhood trauma, psychiatric symptoms, and clinical features of PNES were analyzed.

**Results:**

A total of 35 PNES patients and 34 controls were included in this study. Compared with the controls, the PNES patients reported a higher rate of childhood trauma and more severe psychiatric symptoms. In the PNES patients, early separation from parents was related to more types of childhood trauma and emotional neglect (EN); older age and rural residence during childhood were related to sexual abuse (SA). Moreover, childhood SA and trauma accumulation were correlated with the present psychiatric symptoms. Childhood trauma and rural residence were associated with dissociative symptoms. Separation from parents predicted an earlier PNES onset, whereas childhood SA predicted a later onset. More severe dissociative symptoms were associated with higher seizure frequency.

**Conclusions:**

Childhood trauma is related to the development of PNES and the compromised mental health in PNES patients. This highlights the importance of child protection for preventing psychiatric disorders such as PNES.

**Supplementary Information:**

The online version contains supplementary material available at 10.1186/s42494-024-00180-5.

## Background

Psychogenic non-epileptic seizures (PNES) constitute a range of paroxysmal and involuntary episodic events resembling epileptic seizures that are associated with complex biopsychosocial causes rather than epileptic activity [1–3]. It is a significant neuropsychiatric condition with an estimated prevalence of 2–33/100,000 [4] and an annual incidence of at least 1.5–6.17/100,000 [2]. PNES is often misdiagnosed as epilepsy and affects 20–30% of patients attending epilepsy centers [3].

While the neurobiological mechanisms of PNES remain incompletely understood, the psycho-social risks and associated psychological mechanisms have been well described. Traumatic abuse and life adversity, especially childhood trauma, have been recognized to play crucial roles in PNES [2, 5–7]. In a systematic review of 32 studies, an average of 33.2% of PNES participants reported a history of childhood sexual abuse (SA). In addition, 29.9% of PNES participants across 16 studies reported a history of childhood physical abuse (PA) [6]. Childhood psychological abuse was also found to be a unique predictor for PNES diagnosis [8]. Patients with PNES reported a higher frequency of childhood trauma across all trauma types in the Childhood Trauma Questionnaire compared to patients with epilepsy [9]. A recent review summarized odds ratios of childhood abuse as well as its different categories in PNES patients compared with the epilepsy control group, which mostly fell in the range of 1.8–5.2 [7].

However, there is a discrepancy in the reported prevalence of childhood trauma in PNES patients between Eastern and Western populations. Patients with PNES in Asian countries, including China [10], Iran [11], and India [12, 13], rarely reported a history of SA or PA, ranging from 0 to 12%. However, it is important to note that these studies in Asian populations were based on self-reported information or interviews rather than standardized instruments, which may result in a low proportion of disclosure.

In this study, we set out to study the prevalence of childhood trauma in PNES patients in China, using standardized instruments and cutoff scores for exposure. Furthermore, the correlations of childhood trauma with present psychiatric symptoms and clinical characteristics were explored.

## Methods

### Participants

Patients diagnosed with PNES at the epilepsy monitoring unit of West China Hospital were consecutively recruited from May 2015 to August 2017. The diagnosis of PNES was confirmed by 24-h video-electroencephalogram (VEEG) monitoring: at least one typical seizure-like event recorded during VEEG in the absence of epileptiform activity. The VEEG data were reviewed by two experienced epileptologists to confirm the diagnosis of non-epileptic events. Patients with coexisting epilepsy, or patient who had no epilepsy history but were detected with epileptic seizures or interictal epileptiform discharges during the monitoring were excluded. Sex- and age-matched healthy controls were also recruited.

Participants with any known conditions that might significantly affect their general mental health or impair their understanding of the questionnaires were excluded. Such conditions included illiteracy, cancer, physical disability, and serious neurological/psychiatric disorders, e.g., cognitive deficits or schizophrenia. Healthy controls with any forms of seizure were also excluded.

### Interview and questionnaires

Face-to-face interview of each participant was conducted by a trained neurologist. Demographics including sex, age, and education level and childhood environmental information were collected. Childhood environmental information comprised childhood residence (rural or urban region), original family structure (single- or multi-children family), parental divorce (before the age of 16 years), and history of separation from parents (longer than one year at the preschool age). Clinical data of PNES patients were collected, including the age of PNES onset, duration of PNES, semiology type, recent seizure frequency (per month), and present treatment regimen. The PNES was classified into three types based on the semiology: psychogenic minor motor seizures, psychogenic major motor seizures, or unresponsive seizures [10]. The childhood trauma and psychiatric symptoms (including dissociative symptoms) were evaluated using the Childhood Trauma Questionnaire-Short Form (CTQ-SF), Dissociative Experiences Scale (DES) and Symptom Checklist-90 (SCL-90) questionnaires.

#### CTQ-SF

The CTQ-SF is a 28-item self-administered retrospective inventory designed to measure maltreatment history before 16 years of age. Each item describes one experience in childhood/adolescence and is evaluated by a 5-point rating scale from 1 (Never True) to 5 (Very Often True). The CTQ-SF contains 25 clinical items and three validity items to check for extreme response bias. Clinical items are further classified into five subscales: three for Abuse (Emotional, Physical, and Sexual) and two for Neglect (Emotional and Physical) [14]. The CTQ-SF has been verified as a reliable screening instrument in both clinical and research practices [14]. In addition, the reliability of the Chinese version of CTQ-SF has been validated in a previous study [15].

The cutoff scores for *low-to-moderate* exposure to specific trauma categories were as follows: Physical Abuse (PA) ≥ 8, Sexual Abuse (SA) ≥ 6, Emotional Abuse (EA) ≥ 9, Physical Neglect (PN) ≥ 8, and Emotional Neglect (EN) ≥ 10 [16]. The number of positive trauma types based on the cutoff scores was used as the indicator of trauma accumulation.

#### DES

The 28-item DES is widely used to assess dissociative experience. Both the original and the Chinese versions of the DES have been confirmed to have good reliability and validity [17–19]. Possible response options are based on the frequency (0–100%) that respondents experience various dissociative events without the influence of alcohol or drugs. The DES overall score was calculated as the mean score of the 28 items. Generally, an overall score of ≥ 30 indicates potential dissociative disorders [18, 20].

#### SCL-90

The SCL-90 is a multidimensional self-report symptom inventory evaluating general psychiatric distress in the recent week [21]. The checklist includes 90 questions, with each question scored on a 5-point rating scale, from 1 (not at all) to 5 (extreme). The 90 questions contain a wide array of psychiatric symptoms and fall into nine subscale dimensions: Somatization, Obsessive-Compulsive, Interpersonal-Sensitivity, Depression, Anxiety, Hostility, Phobic-Anxiety, Paranoid Ideation (PAR), and Psychoticism (PSY) [22]. The overall mean score and the means of subscale scores were calculated.

### Statistical analysis

Quantitative data are expressed as the median and interquartile range. Qualitative data are summarized as proportions. Independent samples Mann-Whitney U test was used for analyzing continuous data, and the Chi-square test was used for analyzing categorical data.

**Fig. 1:**
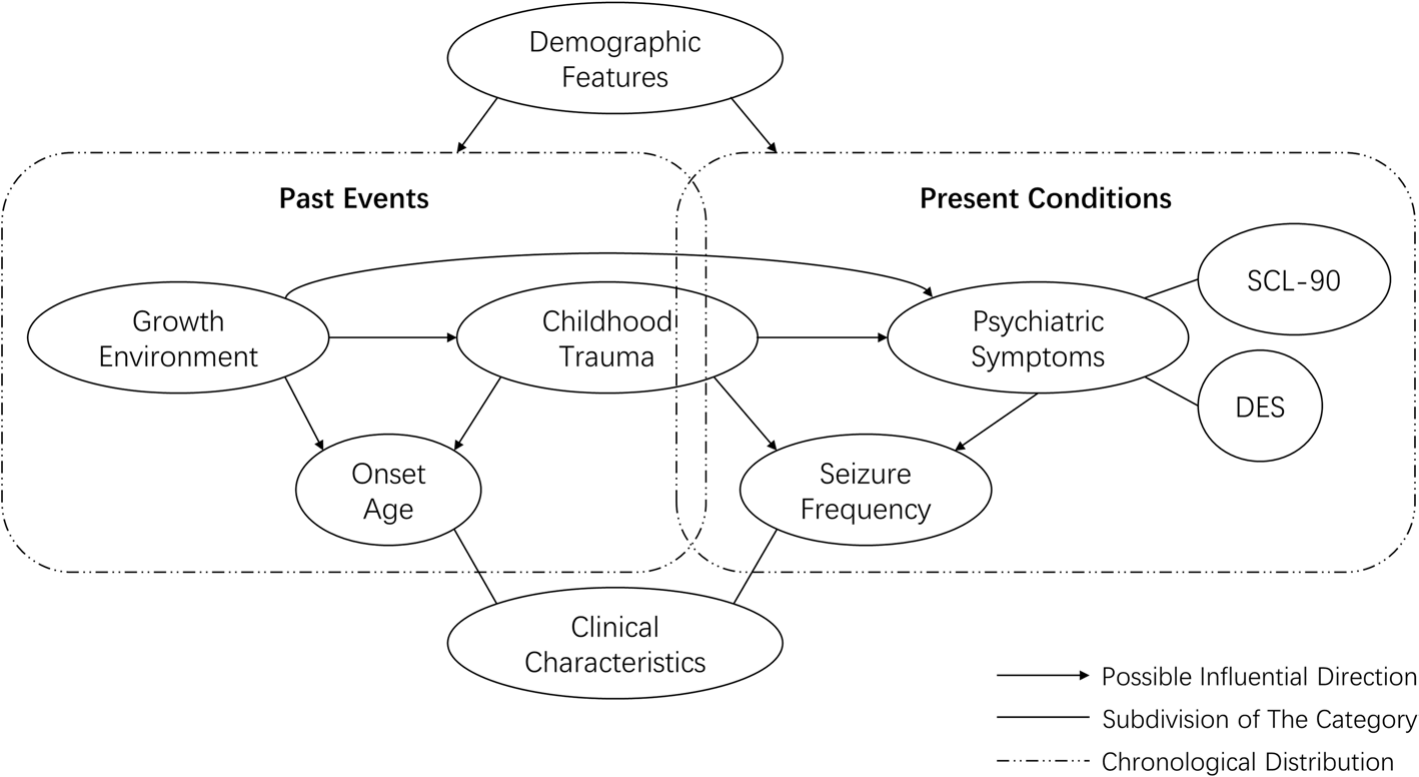
Framework of the study exploring the related factors for childhood trauma, psychiatric symptoms, and clinical characteristics

Childhood trauma was situated within a broader framework encompassing multiple interacting factors (Fig. 1). The childhood environment, childhood trauma, and seizure onset were considered as “past events”. The psychiatric symptoms and seizure frequency were identified as “present conditions”. The childhood trauma consisted of trauma accumulation indicated as the number of positive trauma types, and various types of trauma as measured by the subscales of the CTQ-SF. Psychiatric symptoms included the symptoms measured by the SCL-90 and DES. The direction of influence was as follows: (1) factors in the same chronological category may influence each other; (2) past events may influence the present conditions but not vice versa; and (3) demographic features may influence all other variables. Therefore, the demographic/childhood environmental variables were selected as putative factors for childhood trauma. The demographic/childhood environmental/childhood trauma variables were selected as potential factors for the age of onset of PNES and present psychiatric symptoms. The demographic/childhood trauma/present psychiatric variables were selected as potential influential factors for the seizure frequency.

After determining the putative factors, correlation of the factors with childhood trauma, psychiatric symptoms, and clinical characteristics were analyzed. Pearson’s correlations were used for continuous variables, whereas Spearman correlations were used for ordinal variables. Next, the variables exhibiting a significant correlation underwent multiple linear regression analysis with stepwise selection using entry and exit probabilities of 0.05 and 0.1, respectively. Nonetheless, significantly correlated variables showing little inner connection, such as the age and onset age, were not included in further regression analyses.

Statistical analyses were performed using SPSS version 22.0 (SPSS Inc., Chicago, IL). All tests were two-tailed, and *P* values < 0.05 were considered statistically significant.

## Results

A total of 42 consecutive PNES patients and 37 healthy controls were initially recruited. Seven patients and three controls were excluded due to incomplete questionnaires. Therefore, 35 patients and 34 controls were finally enrolled. All participants provided informed consent, performed the interviews, and completed the questionnaires without missing or ambiguous responses.

### Demographic, childhood environmental and clinical characteristics

The PNES group included 22 females and 13 males. The median age of the PNES group was 16 years (range 14–18 years). There were no significant differences in age, sex, education level, childhood residence, family structure, and rate of parental divorce between the patients and controls. However, the PNES group exhibited a higher rate of separation from parents (48.6%) compared to the controls (23.5%, *P* = 0.03).

The first PNES episodes occurred at a median age of 14 years and psychogenic minor motor seizures were the most frequent seizure type (57.1%). Moreover, the median of PNES frequency was two attacks/month. Most patients (65.7%) received no specific treatment. Among those under treatment, 11 (31.4%) were receiving pharmacotherapy, with similar rates of usage of antiseizure medications, antidepressants, and traditional Chinese medicine. Only two (5.7%) patients received psychotherapy (Table 1).

**Table 1:** Demographics and childhood environmental data of PNES patients and healthy controls, and clinical data of PNES patients

Variables	PNES patients (*n* = 35)	Healthy controls (*n* = 34)	*P* value
Demographics			
Age (years), median (IQR)	16.0 (14.0–18.0)	18.0 (14.0–21.0)	0.250^a^
Sex, female, *n *(%)	22 (62.9)	24 (70.6)	0.496^b^
Education level, *n *(%)			0.348^b^
Primary school	3 (8.6)	3 (8.8)	
Middle school	14 (40.0)	10 (29.4)	
High school	13 (37.1)	10 (29.4)	
College and above	5 (14.3)	11 (32.4)	
Childhood environmental data,* n *(%)			
Childhood rural residence	23 (65.7)	17 (50.0)	0.186^b^
Family structure, single-child family	12 (34.3)	16 (47.1)	0.280^b^
Parental divorce	3 (8.6)	3 (8.8)	0.970^b^
History of parent-child separation	17 (48.6)	8 (23.5)	0.030^b^
Clinical data		NA	NA
Onset age (years), median (IQR)	14.0 (13.0–17.0)		
Disease duration (years), median (IQR)	0.7 (0.3–3.0)		
Semiology type^#^, *n *(%)			
Psychogenic minor motor seizures	20 (57.1)		
Psychogenic major motor seizures	4 (11.4)		
Unresponsive seizures	11 (31.4)		
Seizure frequency per month, median (IQR)	2.0 (0.5-6.0)		
Present treatment, *n *(%)			
Pharmacotherapy	11 (31.4)		
Antiseizure medications	5 (14.3)		
Antidepressants	4 (11.4)		
Traditional Chinese medicine	4 (11.4)		
Psychotherapy	2 (5.7)		
None	23 (65.7)		

^#^For patients with various semiology across different seizures, the typical (most frequent) seizure type is presented for each patient

*Abbreviations:* *PNES* Psychogenic non-epileptic seizures, *IQR* Interquartile range, *NA* Not available

^a^Independent-samples Mann-Whitney U Test

^b^Chi-square Test

### Evaluation of childhood trauma, psychiatric symptoms, and dissociation symptoms

Compared with controls, the PNES patients exhibited significantly higher scores on the CTQ-SF and its PA and PN subscales. Based on the cutoff scores, significantly higher positive rates for PA and PN were observed in the PNES patients. Furthermore, the PNES patients suffered a median of 2 types of trauma, which was significantly higher than the controls (1.5 types, *P* = 0.017). The PNES patients reported more psychiatric symptoms in the SCL-90 questionnaire compared to the controls, with a higher overall mean score of SCL-90 (2.0 vs. 1.3, *P* < 0.001) as reflected in the nine subscales. In addition, the PNES patients also demonstrated more dissociative symptoms with a higher DES median score compared to the controls (25 vs. 13.9, *P* = 0.004) (Table 2).

**Table 2:** Comparison of the scale scores between PNES patients and controls

Variables	PNES patients (*n* = 35)	Healthy controls (*n*=34)	*P* value
CTQ-SF	40.0 (35.0–46.0)	36.5 (32.3–41.3)	0.031^a^
EA	7.0 (6.0–10.0)	6.5 (5.0–8.3)	0.265^a^
EA ≥ 9	12 (34.3)	8 (23.5)	0.325^b^
PA	6.0 (5.0–8.0)	5.0 (5.0–5.3)	0.006^a^
PA ≥ 8	11 (31.4)	3 (8.8)	0.020^b^
SA	5.0 (5.0–6.0)	5.0 (5.0–5.0)	0.078^a^
SA ≥ 6	10 (28.6)	4 (11.8)	0.083^b^
EN	12.0 (9.0–14.0)	11.0 (6.8–13.5)	0.389^a^
EN ≥ 10	22 (62.9)	19 (55.9)	0.555^b^
PN	9.0 (7.0–11.0)	7.0 (6.0–9.0)	0.015^a^
PN ≥ 8	26 (74.3)	15 (44.1)	0.011^b^
Number of trauma types	2.0 (1.0–3.0)	1.5 (0.8–2.0)	0.017^a^
SCL-90	2.0 (1.6–2.6)	1.3 (1.1–1.6)	0.000^a^
SOM	1.8 (1.6–2.3)	1.2 (1.1–1.4)	0.000^a^
O-C	2.2 (1.8–2.9)	1.6 (1.3–2.1)	0.001^a^
INT	1.8 (1.6–2.3)	1.3 (1.1–1.6)	0.000^a^
DEP	2.0 (1.4–2.7)	1.3 (1.1–1.5)	0.000^a^
ANX	2.3 (1.7–3.0)	1.3 (1.1–1.8)	0.000^a^
HOS	2.5 (1.5–3.0)	1.3 (1.0–1.8)	0.000^a^
PHOB	1.9 (1.1–2.7)	1.1 (1.0–1.5)	0.000^a^
PAR	1.7 (1.3–2.2)	1.2 (1.0–1.7)	0.005^a^
PSY	1.5 (1.3–2.1)	1.1 (1.0–1.4)	0.000^a^
DES	25.0 (17.9–36.8)	13.9 (5.5–24.6)	0.004^a^

Data are presented as *n* (%) or median (IQR)

*Abbreviations: PNES* Psychogenic non-epileptic seizures, *IQR* Interquartile range, *STQ-SF* Childhood Trauma Questionnaire-Short Form, *EA* Emotional abuse, *PA* Physical abuse, *SA* Sexual abuse, *EN* Emotional neglect, *PN* Physical neglect, *SCL-90* Symptom Checklist-90, *SOM* Somatization, *O-C* Obsessive–compulsive, *INT* Interpersonal-sensitivity, *DEP* Depression, *ANX* Anxiety, *HOS* Hostility, *PHOB* Phobic-anxiety, *PAR* Paranoid ideation, *PSY* Psychoticism, *DES* Dissociative Experience Scale

^ a^Independent-samples Mann-Whitney U Test

^b^Chi-square test

### Factors related to childhood trauma, psychiatric symptoms, and clinical characteristics

#### Factors related to childhood trauma

The correlations of demographics and childhood environmental variables with the childhood trauma are shown in Table S1. Three regression models were produced for the PNES group (Table 3).

**Table 3:** Factors related to childhood trauma (number of trauma types, SA, and EN) in PNES patients

Dependent variables	Adjusted *R*^2^	Independent variables	Standardized Coefficients (Sβ)	*P* value
Number of trauma types	0.098	History of parent-child separation (no=0, yes=1)	0.353	0.037
SA	0.317	Age	0.523	0.001
		Childhood residence (rural=0, urban=1)	−0.405	0.008
EN	0.126	History of parent-child separation (no=0, yes=1)	0.390	0.021

Multiple regression model with stepwise regression

*Abbreviations: PNES* Psychogenic non-epileptic seizures, *SA* Sexual abuse, *EN* Emotional neglect

The history of separation from parents (Sβ = 0.353, *P* = 0.037) accounted for 9.8% of the variance in the number of trauma types. With SA set as the dependent variable, a two-variable model explained 31.7% of the variance: older age (Sβ = 0.523, *P* = 0.001) and rural residence during childhood (Sβ = −0.405, *P* = 0.008) were related to higher SA scores. In addition, the history of separation from parents (Sβ = 0.390, *P* = 0.021) was the only risk factor explaining 12.6% of the variance in EN. In the control group, no factors were found to be related to childhood trauma.

#### Factors related to the psychiatric symptoms

The correlations of demographic, childhood environmental, and childhood trauma variables with the psychiatric symptoms in PNES patients and controls are shown in Table S2. Regression analyses showed that in the PNES patients, a two-variable model explaining 54% of the variance in the overall SCL-90 score was produced (Table 4). Higher SA scores (Sβ = 0.528, *P* < 0.001) and a higher number of trauma types (Sβ = 0.330, *P* = 0.021) were related to higher SCL-90 scores. SA was included in eight out of nine regression models on each SCL-90 subscale, and the number of trauma types was included in three of the nine models. Moreover, a two-variable model including the number of trauma types (Sβ = 0.437, *P* = 0.005) and the childhood residence (Sβ = −0.325, *P* = 0.033) explained 28.5% of the variance in the DES score of the PNES patients. In the control group, EA was the most relevant factor contributing to the SCL-90 (Sβ = 0.609, *P* < 0.001), explaining 35.1% of the variance in the overall SCL-90 score, and was included in all the regression models on SCL-90 subscales. The DES score of the controls was related to two factors, the number of trauma types (Sβ = 0.366, *P* = 0.024) and SA (Sβ = 0.360, *P* = 0.026).

**Table 4:** Factors related to the psychiatric symptoms in both PNES patients and healthy controls

	Dependent variables	Adjusted R^2^	Independent variables	Standardized Coefficients (Sβ)	*P* value
PNES patients (*n*=35)	SCL-90	0.540	SA	0.528	0.000
			Number of trauma types	0.330	0.021
	SOM	0.385	SA	0.635	0.000
	O-C	0.346	SA	0.463	0.002
			Gender (female=0, male=1)	−0.412	0.006
			Number of trauma types	0.312	0.016
	INT	0.351	SA	0.608	0.000
	DEP	0.539	SA	0.523	0.000
			PA	0.350	0.012
	ANX	0.377	Number of trauma types	0.388	0.020
			SA	0.351	0.034
	HOS	0.246	Number of trauma types	0.518	0.001
	PHOB	0.302	SA	0.466	0.003
			Parental divorce (no=0, yes=1)	0.402	0.009
	PAR	0.467	SA	0.695	0.000
	PSY	0.467	SA	0.695	0.000
	DES	0.285	Number of trauma types	0.437	0.005
			Childhood residence (rural=0, urban=1)	−0.325	0.033
Healthy controls (*n*=34)	SCL-90	0.351	EA	0.609	0.000
	SOM	0.220	EA	0.493	0.003
	O-C	0.220	EA	0.493	0.003
	INT	0.191	EA	0.464	0.006
	DEP	0.223	EA	0.497	0.003
	ANX	0.309	EA	0.575	0.000
	HOS	0.402	EA	0.648	0.000
	PHOB	0.329	EA	0.523	0.001
			Family structure (single-child family=0, multi-child family=1)	0.333	0.026
	PAR	0.302	EA	0.505	0.002
			Family structure (single-child family=0, multi-child family=1)	0.321	0.035
	PSY	0.559	EA	0.674	0.000
			PN	0.277	0.024
	DES	0.322	Number of trauma types	0.366	0.024
			SA	0.360	0.026

Multiple regression model with stepwise regression

*Abbreviations: PNES* Psychogenic non-epileptic seizures, *EA* Emotional abuse, *PA* Physical abuse, *SA* Sexual abuse, *PN* Physical neglect, *SCL-90* Symptom Checklist-90, *SOM* Somatization, *O-C* Obsessive–compulsive, *INT* Interpersonal-sensitivity, *DEP* Depression, *ANX* Anxiety, *HOS* Hostility, *PHOB* Phobic-anxiety, *PAR* Paranoid ideation, *PSY* Psychoticism, *DES* Dissociative Experience Scale

#### Factors related to the clinical characteristics

The correlations of demographics, childhood environment, childhood trauma variables, and psychiatric symptoms with clinical characteristics of PNES are shown in Table S3. Although significant correlations between age/education and onset age were found, such correlations reflect a temporal relationship as opposed to an inner connection. Therefore, age and education were not included in the regression model of the age of onset. As shown in Table 5, results of regression analyses showed that higher SA scores (Sβ = 0.494, *P* = 0.001) were related to later onset, while a history of separation from parents (Sβ = −0.365, *P* = 0.013) was related to earlier onset. These two variables accounted for 34.9% of the variance in the onset age. The seizure frequency was only related to the DES score (Sβ = 0.384, *P* = 0.023), which explained 12.1% of the variance.

**Table 5:** Factors related to the onset age and seizure frequency in PNES patients

Dependent variables	Adjusted *R*^2^	Independent variables	Standardized Coefficients (Sβ)	*P* value
Onset age	0.349	SA	0.494	0.001
		History of parent-child separation (no = 0, yes = 1)	−0.365	0.013
Seizure frequency	0.121	DES	0.384	0.023

Multiple regression model with stepwise regression

*Abbreviations: PNES* Psychogenic non-epileptic seizures, *SA* Sexual abuse, *DES* Dissociative Experience Scale

## Discussion

In this study, the PNES patients reported more childhood trauma and higher severity of various psychiatric symptoms compared with healthy controls. We also identified some factors associated with childhood trauma, psychiatric symptoms, and clinical characteristics in PNES patients or controls. Noteworthily, while an age limit was not predetermined, the patient group exhibited a concentration of younger individuals (14–18 years), possibly attributable to their higher compliance. The definite diagnosis of PNES usually necessitates multiple tests and follow-ups. It is notable that the patients diagnosed via VEEG and subsequently maintained follow-up care at our center (so that we could recruit) tended to be younger individuals. Furthermore, older patients were more likely to decline to participate or incompletely fulfill the interview. Nevertheless, this age composition is consistent with the context of childhood trauma, as older patients may experience pathogenic trauma in adulthood, which is beyond the scope of this study. Moreover, older patients would have less accurate recollections of their childhood experiences compared to younger individuals. The key findings are discussed below.

### Higher prevalence of childhood trauma in PNES patients

Studies in Western countries have reported elevated rates of trauma and abuse in PNES patients [5, 6, 23]. However, studies in Asian populations have reported a relatively low prevalence of trauma [10–13]. The contradictory results indicate potential differences in psychopathological mechanisms underlying PNES between Eastern and Western cultures. However, the discrepancy in trauma prevalence may also be attributed to different rates of disclosure due to different study designs [5]. The present study employed standardized instruments, revealing a higher rate of disclosure and a higher prevalence of childhood trauma (the overall trauma accumulation and the aspects of PA and PN) in PNES patients compared to healthy controls. This supported the correlation between the history of childhood trauma and PNES. The use of questionnaires to determine trauma history improves the detection rate and is recommended in future studies.

“Psychological trauma” was previously considered one of the most common traumatic factors in Chinese PNES patients [10]. In the current study, EA and EN were also frequently found in PNES patients, but the prevalence was similar to that in the controls. Despite the high prevalence of EA/EN, the results suggested that they did not play pivotal roles in the pathogenesis of PNES. In contrast, childhood PA/PN might be more relevant. SA showed no statistically significant difference between PNES patients and controls, but a trend of higher exposure rate to SA was observed in patients compared to controls (28.6% vs. 11.8%). In addition, SA had a significant impact on the psychiatric symptoms of PNES patients, as discussed below. Hence, SA could be considered as a risk factor for PNES in China.

### Higher severity of psychiatric symptoms in PNES patients

Compared with healthy controls [24], PNES patients scored significantly higher on the overall SCL-90 and all its nine subscales, as well as the DES, indicating poorer mental health. These results were in accordance with previous findings. PNES is closely linked with psychological distress. PNES patients have elevated risks of psychiatric comorbidities such as PTSD, depression, anxiety, somatization, and personality disorders [2, 25–28]. In terms of dissociative symptoms, one theoretical approach considers PNES as a dissociative phenomenon characterized by ictal symptoms arising from an impairment in the individual’s ability to synthesize mental contents when challenged with stress or intense emotion [29]. Symptoms of dissociation are often reported by patients with PNES and overlap with other psychological distress [30, 31]. On average, patients with PNES tend to exhibit moderate scores on the DES, which fall slightly below the established cutoff of 30 [29]. Similarly, our PNES group had a median DES score of 25.

### Childhood trauma in PNES patients: higher risk for children living in rural areas and/or experiencing early separation from parents

In the PNES group, risk factors for childhood trauma were found. A high proportion of PNES patients reported a history of early separation from parents. This history was related to more extensive childhood trauma and more severe EN. Most participants reported that their parents were migrant workers who sacrificed family time in favor of financial improvement. In the last 30 years, China has undergone one of the largest rural-to-urban migrations in human history, with many children left behind. Disrupted parent-child relationships predispose the children to non-optimal development [32]. Our results showed that the separation history contributed to a traumatic childhood in PNES patients, which might be one of the side effects of this major societal change. Family functioning mediates the relations between early trauma and later psychological problems [6, 8]. Among various kinds of family dysfunction, this parent-child separation might be particularly pertinent to the Chinese population.

Furthermore, older age and growing up in rural areas were related to more severe SA in PNES patients, with the risk of experiencing childhood SA, which peaks in adolescence [33]. Older age was associated with a greater risk of SA in 18,341 Chinese students, with a mean age of 15.9 years [34]. As our patients had a median age of 16 years, it was plausible that the older individuals in this age group had a higher exposure to SA. However, a previous study in Chinese population reported no significant association between rural residence during childhood and the risk of child SA [24]. Consistently, we also found no association in our control group. Nonetheless, growing up in rural areas bears other unfavorable conditions. Rural students exhibited lower self-esteem and less social support compared to their urban counterparts [35]. Adolescents with unsatisfactory social support showed greater hysterical tendencies [36], yet in SA survivors, supportive relationships strongly predict resilience [37]. Moreover, many people in rural areas of China are still holding improper concepts toward sex and have poor access to sex education [38]. Presumably, SA only contributes to the development of PNES in the presence of inadequate/inappropriate support in rural regions, thereby resulting in the association between rural residence and SA in this PNES population.

### Impact of childhood trauma on mental health: different profiles in patients and controls

Experience of childhood trauma can have long-term effects on the mental health [39]. The present study supported the association between childhood trauma and present psychiatric distress, and revealed different profiles of influential trauma types between PNES patients and healthy controls.

In controls, childhood EA was a significant predictor for high SCL-90 scores, indicating that in the general population, childhood EA resulted in a higher risk of compromised mental health compared to other trauma types. Likewise, a meta-analysis concluded that childhood maltreatment increases the risk of suicidal behavior, with EA exerting the strongest effect [40]. In contrast, a history of SA predicted most psychiatric symptoms in the PNES group. More importantly, the detrimental effects of childhood SA on many areas of mental health are well-documented [41] and may play a unique role in the development of PNES. Childhood SA has been described as a contributory factor in some cases of PNES [6]. In a recent study, the childhood SA reported by participants showed the best screening performance for PNES vs. epilepsy when used as an isolated measure [9]. In addition to SA, the number of trauma types was also an important predictor for some psychiatric symptoms in the PNES patients in this study. Some PNES patients did not report a history of SA, indicating that trauma accumulation might be another risk factor for impaired mental health.

As for dissociative symptoms, the number of trauma types was predictive of the DES score in both groups. Other factors included the rural residence in the PNES group and SA in the control group. These results supported previous literature reporting an association between childhood trauma and dissociation [42, 43]. Moreover, dissociative disorders are more commonly seen in people from lower socioeconomic status and rural areas [44, 45]. In the patient group, rural residence was a more significant predictor of dissociative symptoms than SA, which may be attributed to the additional adversities discussed above.

### Clinical characteristics affected by childhood trauma

Two variables were correlated with the onset age of PNES: SA predicted later onset, and the history of parent-child separation predicted earlier onset. These results possibly reflect two forms of traumatic events typically occurring in different life periods. In this study, the child separation from parents was defined as the individual being not under his/her parents’ tutelage for longer than one year at a preschool age. Therefore, separation-related trauma as defined in this study would occur before six or seven years of age. Additionally, older age was related to higher SA exposure in the patient group. In this study, the parent-child separation and SA tended to occur in different life periods, i.e. before and after puberty. Earlier trauma was shown to promote earlier PNES onset. A previous study reported that SA was associated with earlier PNES onset [46]. The “earlier onset” in that study was 28.5 years compared to the “later onset” of 33.1 years. In comparison, our patients had a median onset age of 14 years. Hence, the discrepancy between the two studies was probably due to the sampling in different age groups.

The PNES frequency was related to only one factor, the DES score. This was in line with the theoretical approach regarding PNES as a dissociative phenomenon [29], as the seizure frequency reflected the severity of PNES, and the DES score reflected the severity of dissociative symptoms.

### Limitations

This study has some limitations. First, the small sample size and the patient age composition limited extrapolation of the conclusion of this study. Our patients were relatively young and might be more representative of patients who developed PNES in the early stage of life. Second, regression analyses were based on a paradigm of chronological distribution, which was putative and contained limitations. For example, past events (childhood trauma) were presumed to influence the present conditions (psychiatric symptoms). However, such chronological distribution was not clearly discrete. As some of the participants were under 16 years old, childhood trauma could still be the “present”. Third, due to the lack of direct inquiry about trauma history, the trauma revealed in this study might only lie in the range reflected by the questionnaire. Lastly, the data were acquired by questionnaire interviews, which may cause recall bias. Despite our attempts to explore the possible causal relationships, the causality should be validated in future longitudinal studies.

## Conclusions

As in many other parts of the world, PNES patients in China experienced more childhood trauma, including PA, PN, and probably SA. Childhood trauma is partly responsible for the development of PNES and the poor mental health of PNES patients. Early parent-child separation due to parental migration might be a risk factor for childhood trauma in the Chinese population and should be addressed in a changing societal context. Proper sex education and social support are particularly needed in rural areas of China. In summary, the results of this study highlight the importance of child protection considering the long-term effects of childhood trauma.

## Supplementary Information

Supplementary Material 1.

## Data Availability

The data supporting the findings of this study are available from the corresponding author upon reasonable request.

## Abbreviations

CTQ-SF: Childhood trauma questionnaire-short form
DES: Dissociative experiences scale
EA: Emotional abuse
EN: Emotional neglect
PA: Physical abuse
PN: Physical neglect
PNES: Psychogenic non-epileptic seizures
SA: Sexual abuse
SCL-90: Symptom checklist-90
Sβ: Standardized coefficients
VEEG: Video-electroencephalogram

## References

[1] Reuber M, Brown RJ. Understanding psychogenic nonepileptic seizures-Phenomenology, semiology and the integrative cognitive model. Seizure. 2017;44:199–205. 27988107 10.1016/j.seizure.2016.10.029

[2] Popkirov S, Asadi-Pooya AA, Duncan R, Gigineishvili D, Hingray C, Miguel Kanner A, et al. The aetiology of psychogenic non-epileptic seizures: risk factors and comorbidities. Epileptic Disorders: Int Epilepsy J Videotape. 2019;21(6):529–47. 10.1684/epd.2019.1107 31843732

[3] Lanzillotti AI, Sarudiansky M, Lombardi NR, Korman GP, D Alessio L. Updated review on the diagnosis and primary management of Psychogenic Nonepileptic Seizure disorders. Neuropsychiatr Dis Treat. 2021;17:1825–38. 34113112 10.2147/NDT.S286710 PMC8187153

[4] Benbadis SR, Allen Hauser W. An estimate of the prevalence of psychogenic non-epileptic seizures. Seizure. 2000;9(4):280–1. 10880289 10.1053/seiz.2000.0409

[5] Beghi M, Cornaggia I, Magaudda A, Perin C, Peroni F, Cornaggia CM. Childhood trauma and psychogenic nonepileptic seizures: a review of findings with speculations on the underlying mechanisms. Epilepsy Behav. 2015;52(Pt A):169–73. 26432009 10.1016/j.yebeh.2015.09.007

[6] Sharpe D, Faye C. Non-epileptic seizures and child sexual abuse: a critical review of the literature. Clin Psychol Rev. 2006;26(8):1020–40. 16472897 10.1016/j.cpr.2005.11.011

[7] Jones LL, Rickards H. History of abuse and psychogenic nonepileptic seizures: a systematic review. Seizure. 2021;92:200–4. 34555802 10.1016/j.seizure.2021.09.009

[8] Salmon P, Al-Marzooqi SM, Baker G, Reilly J. Childhood family dysfunction and associated abuse in patients with nonepileptic seizures: towards a causal model. Psychosom Med. 2003;65(4):695–700. 12883124 10.1097/01.psy.0000075976.20244.d8

[9] Yang T, Roberts C, Winton-Brown T, Lloyd M, Kwan P, O’Brien TJ, et al. Childhood trauma in patients with epileptic vs nonepileptic seizures. Epilepsia. 2022;64(1):184–95. 36300720 10.1111/epi.17449 PMC10100454

[10] An DM, Wu XT, Yan B, Mu J, Zhou D. Clinical features of psychogenic nonepileptic seizures: a study of 64 cases in southwest China. Epilepsy Behav. 2010;17(3):408–11. 20149757 10.1016/j.yebeh.2010.01.003

[11] Asadi-Pooya AA, Emami Y, Emami M. Psychogenic non-epileptic seizures in Iran. Seizure. 2014;23(3):175–7. 24315495 10.1016/j.seizure.2013.11.005

[12] Lazarus JP, Bhatia M, Shukla G, Padma MV, Tripathi M, Shrivastava AK, et al. A study of nonepileptic seizures in an Indian population. Epilepsy Behav. 2003;4(5):496–9. 14527490 10.1016/s1525-5050(03)00118-5

[13] Sawant NS, Umate MS. Dissociation, stressors, and coping in patients of psychogenic nonepileptic seizures. Indian J Psychol Med. 2020;43(6):479–84. 35210675 10.1177/0253717620956460 PMC8826194

[14] Bernstein DP, Stein JA, Newcomb MD, Walker E, Pogge D, Ahluvalia T, et al. Development and validation of a brief screening version of the Childhood Trauma Questionnaire. Child Abuse Negl. 2003;27(2):169–90. 12615092 10.1016/s0145-2134(02)00541-0

[15] Zhao XF, Zhang YL, Li LF, Zhou YF, Li HZ, Yang SC. Reliability and validity of the Chinese version of childhood trauma questionnaire. Chin J Clin Rehabilitation. 2005;9(20):105–7.

[16] Tietjen GE, Brandes JL, Peterlin BL, Eloff A, Dafer RM, Stein MR, et al. Childhood maltreatment and migraine (part I). Prevalence and adult revictimization: a multicenter headache clinic survey. Headache. 2010;50(1):20–31. 19845782 10.1111/j.1526-4610.2009.01556.x

[17] Fang L, Liu XH. Detection of the reliability and validity of dissociative experience scale II. Chin J Clin Rehabilitation. 2006;10(42):1–4.

[18] Carlson EB, Putnam FW, Ross CA, Torem M, Coons P, Dill DL, et al. Validity of the dissociative experiences Scale in screening for multiple personality disorder: a multicenter study. Am J Psychiatry. 1993;150(7):1030–6. 8317572 10.1176/ajp.150.7.1030

[19] Bernstein EM, Putnam FW. Development, reliability, and validity of a dissociation scale. J Nerv Ment Dis. 1986;174(12):727–35. 3783140 10.1097/00005053-198612000-00004

[20] van Ijzendoorn MH, Schuengel C. The measurement of dissociation in normal and clinical populations: Meta-analytic validation of the dissociative experiences Scale (DES). Clin Psychol Rev. 1996;16(5):365–82.

[21] Derogatis LR, Lipman RS, Covi L. SCL-90: an outpatient psychiatric rating scale–preliminary report. Psychopharmacol Bull. 1973;9(1):13–28. 4682398

[22] Derogatis LR, Cleary PA. Factorial invariance across gender for the primary symptom dimensions of the SCL-90. Br J Soc Clin Psychol. 1977;16(4):347–56. 588890 10.1111/j.2044-8260.1977.tb00241.x

[23] Fiszman A, Alves-Leon SV, Nunes RG, D’Andrea I, Figueira I. Traumatic events and posttraumatic stress disorder in patients with psychogenic nonepileptic seizures: a critical review. Epilepsy Behav. 2004;5(6):818–25. 15582828 10.1016/j.yebeh.2004.09.002

[24] Chen J, Dunne MP, Han P. Child sexual abuse in China: a study of adolescents in four provinces. Child Abuse Negl. 2004;28(11):1171–86. 15567022 10.1016/j.chiabu.2004.07.003

[25] Tojek TM, Lumley M, Barkley G, Mahr G, Thomas A. Stress and other psychosocial characteristics of patients with psychogenic nonepileptic seizures. Psychosomatics. 2000;41(3):221–6. 10849454 10.1176/appi.psy.41.3.221

[26] Diprose W, Sundram F, Menkes DB. Psychiatric comorbidity in psychogenic nonepileptic seizures compared with epilepsy. Epilepsy Behav. 2016;56:123–30. 26874243 10.1016/j.yebeh.2015.12.037

[27] Mökleby K, Blomhoff S, Malt UF, Dahlström A, Tauböll E, Gjerstad L. Psychiatric comorbidity and hostility in patients with psychogenic nonepileptic seizures compared with somatoform disorders and healthy controls. Epilepsia. 2002;43(2):193–8. 11903468 10.1046/j.1528-1157.2002.20901.x

[28] Scévola L, Wolfzun C, Sarudiansky M, Pico MMA, Ponieman M, Stivala EG, et al. Psychiatric disorders, depression and quality of life in patients with psychogenic non-epileptic seizures and drug resistant epilepsy living in Argentina. Seizure. 2021;92:174–81. 34536854 10.1016/j.seizure.2021.09.004

[29] Brown RJ, Reuber M. Psychological and psychiatric aspects of psychogenic non-epileptic seizures (PNES): a systematic review. Clin Psychol Rev. 2016;45:157–82. 27084446 10.1016/j.cpr.2016.01.003

[30] Cohen ML, Testa SM, Pritchard JM, Zhu J, Hopp JL. Overlap between dissociation and other psychological characteristics in patients with psychogenic nonepileptic seizures. Epilepsy Behav. 2014;34:47–9. 24681385 10.1016/j.yebeh.2014.03.001

[31] Kuyk J, Van Dyck R, Spinhoven P. The case for a dissociative interpretation of pseudoepileptic seizures. J Nerv Ment Dis. 1996;184(8):468–74. 8752075 10.1097/00005053-199608000-00003

[32] Wang L, Mesman J. Child Development in the Face of Rural-to-Urban Migration in China: a Meta-Analytic Review. Perspect Psychol Sci. 2015;10(6):813–31. 26581737 10.1177/1745691615600145

[33] Bebbington PE, Jonas S, Brugha T, Meltzer H, Jenkins R, Cooper C, et al. Child sexual abuse reported by an English national sample: characteristics and demography. Soc Psychiatry Psychiatr Epidemiol. 2011;46(3):255–62. 20544176 10.1007/s00127-010-0245-8

[34] Chan KL, Yan E, Brownridge DA, Ip P. Associating child sexual abuse with child victimization in China. J Pediatr. 2013;162(5):1028–34. 23219443 10.1016/j.jpeds.2012.10.054

[35] Zhang J, Qi Q, Delprino RP. Psychological health among Chinese college students: a rural/urban comparison. J Child Adolesc Ment Health. 2017;29(2):179–86. 28974168 10.2989/17280583.2017.1345745

[36] Cheng Q, Xie L, Hu Y, Hu J, Gao W, Lv Y, et al. Gender differences in the prevalence and impact factors of hysterical tendencies in adolescents from three eastern Chinese provinces. Environ Health Prev Med. 2018;23(1):5. 29415649 10.1186/s12199-018-0695-2 PMC5803911

[37] Sanjeevi J, Houlihan D, Bergstrom KA, Langley MM, Judkins J. A review of child sexual abuse: impact, risk, and Resilience in the Context of Culture. J Child Sex Abus. 2018;27(6):622–41. 10.1080/10538712.2018.1486934 30064308

[38] Wang B, Davidson P. Sex, lies, and videos in rural China: a qualitative study of women. J Sex Res. 2006;43(3):227–35. 17599245 10.1080/00224490609552321

[39] Forkey H, Szilagyi M. Foster care and healing from complex childhood trauma. Pediatr Clin North Am. 2014;61(5):1059–72. 25242716 10.1016/j.pcl.2014.06.015

[40] Liu J, Fang Y, Gong J, Cui X, Meng T, Xiao B, et al. Associations between suicidal behavior and childhood abuse and neglect: a meta-analysis. J Affect Disord. 2017;220:147–55. 28623759 10.1016/j.jad.2017.03.060

[41] Caro P, Turner W, Caldwell DM, Macdonald G. Comparative effectiveness of psychological interventions for treating the psychological consequences of sexual abuse in children and adolescents: a network meta-analysis. Cochrane Database Syst Reviews. 2023;2023:6. 10.1002/14651858.CD013361.pub2 PMC10243720 37279309

[42] Foote B, Smolin Y, Kaplan M, Legatt ME, Lipschitz D. Prevalence of dissociative disorders in psychiatric outpatients. Am J Psychiatry. 2006;163(4):623–9. 16585436 10.1176/ajp.2006.163.4.623

[43] Xiao Z, Yan H, Wang Z, Zou Z, Xu Y, Chen J, et al. Trauma and dissociation in China. Am J Psychiatry. 2006;163(8):1388–91. 16877651 10.1176/ajp.2006.163.8.1388

[44] Tabassum K, Farooq S. Sociodemographic features, affective symptoms and family functioning in hospitalized patients with dissociative disorder (convulsion type). J Pak Med Assoc. 2007;57(1):23–6. 17319415

[45] Reddy LS, Patil NM, Nayak RB, Chate SS, Ansari S. Psychological dissection of patients having dissociative disorder: a cross-sectional study. Indian J Psychol Med. 2018;40(1):41–6. 29403129 10.4103/IJPSYM.IJPSYM_237_17 PMC5795678

[46] Selkirk M, Duncan R, Oto M, Pelosi A. Clinical differences between patients with nonepileptic seizures who report antecedent sexual abuse and those who do not. Epilepsia. 2008;49(8):1446–50. 18410361 10.1111/j.1528-1167.2008.01611.x

