# Supplementary material for: Role of childhood trauma in psychogenic non-epileptic seizures: a report from China
Source: Acta Epileptol. 2025 Jan 6;7:4. doi: 10.1186/s42494-024-00180-5 (PMC11960396; doi:10.1186/s42494-024-00180-5)
Supplement: Supplementary file 1 — Supplementary Material 1. [file 42494_2024_180_MOESM1_ESM.docx]

Table S1. Correlations of demographic and childhood environmental variables with childhood trauma (number of trauma types and each type of trauma) in both PNES patients and controls.

|  | Potentially related factors to the childhood trauma | Childhood trauma | | | | | |
| --- | --- | --- | --- | --- | --- | --- | --- |
|  |  | Number of trauma types | EA | PA | SA | EN | PN |
| PNES Patients (*n*=35) | Sex (female=0, male=1)^b^ | −0.03 | -0.17 | -0.07 | 0.02 | 0.16 | -0.02 |
|  | Age^a^ | 0.02 | -0.08 | 0.03 | 0.45** | -0.14 | -0.07 |
|  | Education (primary school=1, middle school=2, high school=3, college and above=4)^b^ | −0.18 | 0.16 | -0.17 | -0.05 | -0.24 | -0.13 |
|  | Childhood residence (rural=0, urban=1)^b^ | −0.11 | 0.12 | -0.04 | -0.34* | -0.19 | 0.02 |
|  | Family structure (single-child family=0, multi-child family=1)^b^ | 0.15 | -0.11 | 0.20 | 0.34* | 0.11 | 0.01 |
|  | Parental divorce (no=0, yes=1)^b^ | 0.02 | 0.04 | -0.30 | -0.01 | 0.13 | -0.03 |
|  | History of parent-child separation (no=0, yes=1)^b^ | 0.36* | 0.14 | 0.22 | 0.03 | 0.36* | 0.15 |
| Healthy controls (*n*=34) | Sex (female=0, male=1)^b^ | 0.03 | 0.09 | -0.03 | -0.02 | 0.02 | -0.18 |
|  | Age^a^ | −0.17 | -0.15 | -0.28 | -0.10 | -0.20 | -0.04 |
|  | Education (primary school=1, middle school=2, high school=3, college and above=4)^b^ | −0.07 | -0.14 | -0.10 | 0.06 | -0.04 | -0.01 |
|  | Childhood residence (rural=0, urban=1)^b^ | 0.07 | 0.21 | 0.11 | 0.02 | 0.63 | -0.11 |
|  | Family structure (single-child family=0, multi-child family=1)^b^ | −0.17 | -0.08 | -0.03 | -0.04 | 0.03 | -0.04 |
|  | Parental divorce (no=0, yes=1)^b^ | −0.03 | -0.14 | 0.11 | -0.11 | -0.02 | -0.03 |
|  | History of parent-child separation (no=0, yes=1)^b^ | 0.16 | 0.12 | 0.05 | -0.20 | 0.06 | 0.05 |
| ^a^ Pearson's correlation was applied.  ^b^ Spearman correlation was applied.  * *P* < 0.05  ** *P* < 0.01  Abbreviations: PNES: psychogenic non-epileptic seizures; EA: emotional abuse, PA: physical abuse, SA: sexual abuse, EN: emotional neglect, PN: physical neglect. | | | | | | | |

Table S2. Correlations of demographic, childhood environmental, and childhood trauma variables with the psychiatric symptoms in both PNES patients and controls.

|  | Potentially related factors to psychiatric symptoms | Psychiatric Symptoms | | | | | | | | | | |
| --- | --- | --- | --- | --- | --- | --- | --- | --- | --- | --- | --- | --- |
|  |  | SCL-90 | SOM | O-C | INT | DEP | ANX | HOS | PHOB | PAR | PSY | DES |
| PNES patients (*n*=35) | Sex (female=0, male=1)^b^ | −0.24 | −0.09 | −0.39* | −0.23 | −0.28 | −0.17 | −0.07 | −0.16 | −0.17 | −0.05 | -0.13 |
|  | Age^a^ | 0.30 | 0.15 | 0.27 | 0.23 | 0.34* | 0.17 | 0.21 | 0.07 | 0.28 | 0.38* | -0.07 |
|  | Education (primary school=1, middle school=2, high school=3, college and above=4)^b^ | −0.26 | −0.20 | 0.11 | −0.33 | −0.10 | −0.25 | 0.01 | −0.12 | 0.02 | 0.05 | -0.03 |
|  | Childhood residence (rural=0, urban=1)^b^ | −0.10 | −0.11 | 0.01 | −0.16 | −0.19 | −0.04 | 0.09 | −0.11 | −0.21 | −0.12 | -0.41* |
|  | Family structure (single-child family=0, multi-child family=1)^b^ | 0.18 | 0.08 | 0.32 | 0.16 | 0.30 | 0.14 | −0.01 | 0.17 | 0.21 | 0.26 | 0.41* |
|  | Parental divorce (no=0, yes=1)^b^ | 0.18 | 0.04 | 0.02 | −0.03 | 0.16 | 0.26 | 0.23 | 0.34* | 0.11 | 0.15 | 0.08 |
|  | History of parent-child separation (no=0, yes=1)^b^ | 0.03 | 0.14 | −0.06 | 0.05 | −0.07 | 0.04 | 0.05 | −0.02 | 0.07 | −0.13 | -0.02 |
|  | Number of trauma types^a^ | 0.60** | 0.49** | 0.44** | 0.49** | 0.60** | 0.57** | 0.52** | 0.36* | 0.54** | 0.51** | 0.47** |
|  | EA^a^ | 0.37* | 0.27 | 0.30 | 0.34* | 0.43* | 0.46** | 0.33 | 0.10 | 0.21 | 0.23 | 0.11 |
|  | PA^a^ | 0.50** | 0.30 | 0.43** | 0.40* | 0.59** | 0.55** | 0.40* | 0.19 | 0.37* | 0.39* | 0.33 |
|  | SA^a^ | 0.70** | 0.64** | 0.46** | 0.61** | 0.69** | 0.55** | 0.43** | 0.43* | 0.69** | 0.70** | 0.47** |
|  | EN^a^ | 0.23 | 0.17 | 0.13 | 0.23 | 0.17 | 0.17 | 0.18 | 0.28 | 0.18 | 0.25 | 0.15 |
|  | PN^a^ | 0.30 | 0.14 | 0.33 | 0.07 | 0.34* | 0.42* | 0.19 | 0.13 | 0.14 | 0.26 | 0.19 |
| Healthy controls (*n*=34) | Sex (female=0, male=1)^b^ | −0.14 | −0.08 | −0.09 | −0.12 | −0.01 | −0.28 | 0.00 | −0.24 | −0.15 | −0.20 | 0.05 |
|  | Age^a^ | −0.12 | −0.08 | −0.08 | −0.12 | −0.02 | −0.09 | −0.16 | −0.13 | −0.23 | −0.15 | -0.26 |
|  | Education (primary school=1, middle school=2, high school=3, college and above=4)^b^ | −0.24 | −0.20 | −0.06 | −0.23 | −0.05 | −0.24 | −0.22 | −0.14 | −0.32 | −0.22 | -0.34* |
|  | Childhood residence (rural=0, urban=1)^b^ | 0.10 | 0.04 | 0.17 | −0.15 | 0.19 | 0.17 | 0.21 | −0.22 | −0.14 | 0.08 | -0.19 |
|  | Family structure (single-child family=0, multi-child family=1)^b^ | 0.11 | −0.02 | 0.04 | 0.19 | 0.00 | 0.08 | −0.07 | 0.33* | 0.40* | 0.14 | 0.05 |
|  | Parental divorce (no=0, yes=1)^b^ | −0.25 | −0.16 | −0.22 | −0.09 | −0.28 | −0.14 | −0.15 | −0.17 | −0.29 | −0.10 | -0.28 |
|  | History of parent-child separation (no=0, yes=1)^b^ | −0.15 | −0.03 | −0.08 | −0.07 | −0.18 | −0.09 | −0.17 | −0.18 | −0.29 | −0.25 | -0.13 |
|  | Number of trauma types^a^ | 0.25 | 0.01 | 0.18 | 0.22 | 0.15 | 0.35* | 0.28 | 0.19 | 0.23 | 0.47** | 0.47** |
|  | EA^a^ | 0.61** | 0.54** | 0.49** | 0.46** | 0.50** | 0.58** | 0.65** | 0.51** | 0.49** | 0.71** | 0.31 |
|  | PA^a^ | 0.23 | 0.18 | 0.19 | 0.17 | 0.17 | 0.16 | 0.44** | 0.04 | 0.31 | 0.31 | 0.41** |
|  | SA^a^ | 0.05 | 0.03 | 0.04 | −0.14 | −0.04 | 0.03 | 0.21 | −0.12 | 0.11 | 0.26 | 0.50** |
|  | EN^a^ | 0.30 | 0.06 | 0.24 | 0.29 | 0.30 | 0.38* | 0.20 | 0.33 | 0.38* | 0.31 | 0.22 |
|  | PN^a^ | 0.07 | −0.03 | −0.06 | −0.04 | 0.01 | 0.19 | 0.08 | 0.01 | 0.08 | 0.38* | 0.45** |
| ^a^ Pearson's correlation was applied.  ^b^ Spearman correlation was applied.  * *P* < 0.05  ** *P* < 0.01  Abbreviations: PNES: psychogenic non-epileptic seizures; EA: emotional abuse, PA: physical abuse, SA: sexual abuse, EN: emotional neglect, PN: physical neglect; SCL-90: Symptom Checklist-90, SOM: somatization, O-C: obsessive–compulsive, INT: interpersonal-sensitivity, DEP: depression, ANX: anxiety, HOS: hostility, PHOB: phobic-anxiety, PAR: paranoid ideation, PSY: psychoticism; DES: Dissociative Experience Scale. | | | | | | | | | | | | |

Table S3. Analyses of related factors for the clinical characteristics (onset age and seizure frequency) in PNES patients (*n*=35).

| Potential factors related to onset age and seizure frequency | Onset age | Seizure frequency |
| --- | --- | --- |
| Sex (female=0, male=1)^b^ | 0.17 | 0.07 |
| Age^a#^ | 0.77** | −0.02 |
| Education (primary school=1, middle school=2, high school=3, college and above=4)^b#^ | 0.36* | −0.50** |
| Childhood residence (rural=0, urban=1)^b^ | 0.08 | − |
| Family structure (single-child family=0, multi-child family=1)^b^ | −0.17 | − |
| Parental divorce (no=0, yes=1)^b^ | −0.14 | − |
| History of parent-child separation (no=0, yes=1)^b^ | −0.38* | − |
| Number of trauma types^a^ | −0.03 | 0.13 |
| EA^a^ | 0.01 | −0.18 |
| PA^a^ | 0.09 | 0.09 |
| SA^a^ | 0.50** | 0.22 |
| EN^a^ | −0.18 | 0.02 |
| PN^a^ | −0.23 | 0.29 |
| SCL-90^a^ | − | 0.22 |
| SOM^a^ | − | 0.34* |
| O-C^a^ | − | −0.04 |
| INT^a^ | − | 0.29 |
| DEP^a^ | − | 0.05 |
| ANX^a^ | − | 0.18 |
| HOS^a^ | − | 0.21 |
| PHOB^a^ | − | 0.32 |
| PAR^a^ | − | 0.31 |
| PSY^a^ | − | 0.18 |
| DES^a^ | − | 0.38* |
| ^a^ Pearson's correlation was applied.  ^b^ Spearman correlation was applied.  * *P* < 0.05  ** *P* < 0.01  ^#^ Age and education were not included in further regression analyses of the onset age.  Abbreviations: PNES: psychogenic non-epileptic seizures; EA: emotional abuse, PA: physical abuse, SA: sexual abuse, EN: emotional neglect, PN: physical neglect; SCL-90: Symptom Checklist-90, SOM: somatization, O-C: obsessive–compulsive, INT: interpersonal-sensitivity, DEP: depression, ANX: anxiety, HOS: hostility, PHOB: phobic-anxiety, PAR: paranoid ideation, PSY: psychoticism; DES: Dissociative Experience Scale. | | |
